# Efficacy of Pea Protein Supplementation in Combination with a Resistance Training Program on Muscle Performance in a Sedentary Adult Population: A Randomized, Comparator-Controlled, Parallel Clinical Trial

**DOI:** 10.3390/nu16132017

**Published:** 2024-06-26

**Authors:** Ruma G. Singh, Laetitia Guérin-Deremaux, Catherine Lefranc-Millot, Caroline Perreau, David C. Crowley, Erin D. Lewis, Malkanthi Evans, Marc Moulin

**Affiliations:** 1 KGK Science Inc., London, ON N6B 3L1, Canada; rsingh@kgkscience.com (R.G.S.); dcrowley@kgkscience.com (D.C.C.); elewis@kgkscience.com (E.D.L.); mevans23@uwo.ca (M.E.); 2 Life Sciences R&D, Roquette, 62136 Lestrem, France; laetitia.guerin-deremaux@roquette.com (L.G.-D.); caroline.perreau@roquette.com (C.P.); catherine.lefranc-millot@roquette.com (C.L.-M.); 3 Department of Biochemistry, Western University, London, ON N6A 3K7, Canada

**Keywords:** body composition, body strength, handgrip strength, muscle mass, pea protein, whey protein

## Abstract

Animal-sourced whey protein (WPr) is the most popular protein supplement among consumers and has been shown to improve muscle mass and strength. However, due to allergies, dietary restrictions/personal choices, and growing demand, alternative protein sources are warranted. Sedentary adults were randomized to pea protein (PPr) or WPr in combination with a weekly resistance training program for 84 days. Changes in whole-body muscle strength (WBMS) including handgrip, lower body, and upper body strength, body composition, and product perception were assessed. The safety outcomes included adverse events, vital signs, clinical chemistry, and hematology. There were no significant differences in the change in WBMS, muscle mass, or product perception and likability scores between the PPr and WPr groups. The participants supplemented with PPr had a 16.1% improvement in WBMS following 84 days of supplementation (*p* = 0.01), while those taking WPr had an improvement of 11.1% (*p* = 0.06). Both study products were safe and well-tolerated in the enrolled population. Eighty-four days of PPr supplementation resulted in improvements in strength and muscle mass comparable to WPr when combined with a resistance training program in a population of healthy sedentary adults. PPr may be considered as a viable alternative to animal-sourced WPr without sacrificing muscular gains and product enjoyment.

## 1. Introduction

Sedentary behavior is associated with increased body fat mass and loss of muscle mass and strength [1]. The combination of adequate dietary protein intake and resistance exercise has been shown to have a positive effect on muscle function [2,3]. The Recommended Dietary Allowance (RDA), the daily minimum intake, for dietary protein is 0.83 g (g)/kilogram (kg) body weight (bw)/day for healthy adults with minimal physical activity [4]. However, individuals participating in physical activity require additional protein consumption to meet the demands of exercise and to sustain their physical health and function [3]. Therefore, adults who routinely complete moderate physical activity are advised to increase their dietary protein intake to 1.2–2.0 g/kg bw/day [5]. Further, a sedentary behavior and poor diet quality negatively impact the efficiency of essential amino acids (EAAs) to stimulate protein metabolism, requiring the consumption of more protein or high-quality proteins [6]. Given the importance of adequate protein intake for optimal protein metabolism and muscle performance, protein powder may be an effective and convenient dietary supplement to fulfill the required daily protein intake in healthy individuals who are exercising.

Whey protein (WPr), one of the most widely used animal-sourced proteins, provides health benefits as it contains lactose, minerals, vitamins, and soluble proteins [7,8] and has been shown to enhance muscle mass and strength. A meta-analysis of 11 randomized clinical trials demonstrated that WPr combined with a resistance exercise program significantly improved the muscle strength in healthy individuals when compared to carbohydrate placebos and exercise [9]. Despite the known benefits of WPr, there is growing interest in plant-based protein alternatives as these products can be consumed by individuals who avoid animal-sourced proteins due to allergies, dietary restrictions, and/or personal dietary choices [10]. Furthermore, rising awareness of the environmental impacts of animal food production and associations with improvements in health and reductions in all-cause mortality [11,12] have led to increases in the global consumption of and demand for plant-based proteins. Further, there are noteworthy differences between plant- and animal-based proteins that should be considered, including differences in digestibility [13] and/or amounts of certain EAAs [14], which are important factors for muscle health and function [15]. However, emerging evidence suggests that both types of proteins may have similar effects on protein synthesis [16].

Pea protein (PPr) is gaining popularity, with 80% of plant-based protein powders containing PPr [17]. Few studies have examined the equivalence between PPr and WPr in relation to muscle performance in sedentary adults. A study of non-athletic and non-obese males reported no difference in exercise-induced performance, muscle damage, and soreness after 5 days of adding PPr into their diet compared to WPr [18]. However, the study was conducted over a short supplementation period of 5 days, providing the rationale for further investigation of the long-term effects of PPr on muscle function and performance. Another study demonstrated that PPr or WPr supplementation in participants undergoing a 12-week resistance training program significantly increased their bicep muscle thickness compared to a placebo [19]. Thus, PPr may be an alternative protein source to improve muscle function in a sedentary population. The purpose of this study was to investigate the functional equivalence of PPr compared to WPr in relation to the muscular performance in a healthy, sedentary adult population who participated in a resistance training program.

## 2. Materials and Methods

### 2.1. Study Design and Ethical Approval

**Figure 1:**
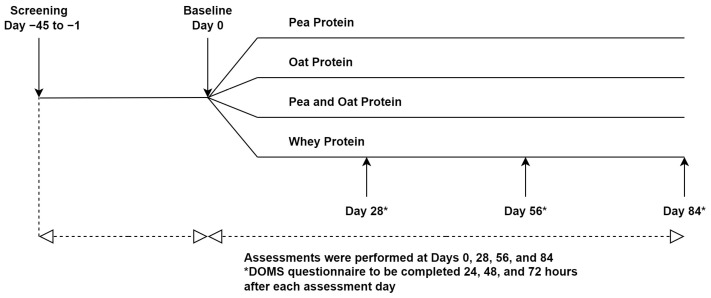
Study Design.

This study was a randomized triple-blind, comparator-controlled, parallel clinical trial conducted at KGK Science Inc. (London, ON, Canada) from April 2021 to April 2022. Research Ethics Board (REB) approval was granted on 26 February 2021 by Advarra, Aurora, Ontario (Pro00049904). The trial followed the CONSORT guidelines for randomized controlled trials (Table S1). The study consisted of an 84-day supplementation period in which participants were randomized to receive one of three plant-based proteins (PPr, Pea, and Oat Protein Powder (POPr), Oat Protein Powder (OPr)) or WPr (clinicaltrials.gov ID: NCT04814225) (Figure 1). The current manuscript reports the findings of PPr and WPr groups only. Informed consent was obtained from the participants prior to performing any study procedures.

### 2.2. Study Population

The participants were adults between 30 and 59 years of age with a sedentary lifestyle, defined as not engaging in more than 60 min of regular moderate to vigorous exercise per week [20,21,22], a waist circumference of <102 cm for men and <88 cm for women and a self-reported stable body weight for three months prior to the baseline. The participants agreed to refrain from taking nonsteroidal anti-inflammatory drugs for 24 h prior to and 72 h after the study visits and maintain their daily caloric intake. The exclusion criteria were: individuals with an allergy, sensitivity, or intolerance to the study products, women who were pregnant, breast feeding, or planning to become pregnant during the study, adherence to a specific diet (e.g., vegan, ketogenic), those who engaged in regular and structured resistance training for ≥2× times per week as determined by the Medical Director (MD), those who had any medical condition or metal implants that would interfere with their ability to complete the physical strength testing, exercise program, or Dual-energy X-ray Absorption (DXA) scans, and those who used concomitant medications, supplements, foods, or drinks that would confound the study outcomes. 

### 2.3. Investigational Products and Comparator

The investigational product (IP) was a PPr powder (NUTRALYS^®^ S85 Plus, ROQUETTE, Vic-sur-Aisne, France) provided in two flavors (vanilla contained 20.0 g of pea protein; chocolate contained 22.5 g of pea protein). The comparator was WPr powder (Whey protein Isolate, Glanbia Nutritionals Inc., Richfield, ID, USA) provided in the same two flavors (vanilla contained 20.0 g of whey protein isolate; chocolate contained 21.1 g of whey protein isolate).

The participants were instructed to mix the study product with 250 mL of room temperature water in a provided shaker bottle once per day for 84 days. The participants were required to consume the product immediately following mixing. On exercise days (see Section Resistance Training Program), the participants were expected to consume the IP after exercise and at approximately the same time as on the days without exercise. The clinic staff instructed the participants to save all unused and open packages and return them for the determination of compliance. If a dose was missed, the participants were instructed to consume the product as soon as they remembered, if within the same day. The participants were instructed not to exceed more than one dose per day. The timing of each daily dose and any missed doses were to be recorded in their study diary.

### 2.4. Randomization and Blinding

A blinded investigator assigned each participant with a randomization number derived from the randomization list (www.randomization.com; accessed on 31 March 2021). The investigators, study personnel, and participants were blinded to the products. The products were sealed in identically appearing sachets, ensuring allocation concealment, and were labeled per the requirements of the International Council for Harmonisation of Technical Requirements for Pharmaceuticals for Human Use-Good Clinical Practice (ICH-GCP) guideline and applicable local regulatory guidelines.

### 2.5. Study Outcomes

The primary outcome was the difference in the change in the composite whole-body muscle strength (WBMS) from the baseline at day 84 between the study groups. The secondary outcomes were the differences in the change in individual muscle strength (upper body, lower body, handgrip), endurance performance, quality of life (QoL), exercise recovery, C-reactive protein (CRP), and product tolerability (as assessed by the modified Gastrointestinal Symptoms Rating scale (GSRS) from the baseline at days 28, 56, and 84. The other secondary outcomes included the differences in the product perception at day 84, change in the body composition from the baseline at day 84, and change in immune function (as assessed via white blood cell (WBC), lymphocyte, and neutrophil counts) from screening at day 84. The safety was assessed via the incidence of adverse events (AEs), and the changes in vital signs, clinical chemistry, and hematology at day 84.

### 2.6. Study Assessments

#### 2.6.1. Muscle Strength

The upper body and lower body strength was assessed using an isometric handheld dynamometer (JTECH Medical, AA 104 REV. U, Midvale, UT, USA) and the handgrip strength was assessed using a hand dynamometer (JAMAR Hydraulic Hand Dynamometer, Item# 081028935, Warrenville, IL, USA). For the assessment of the upper body strength, the participants laid down with their elbow positioned at 90° and completed a series of isometric exertions while a dynamometer was held on their arm proximal to the ulnar head or proximal to the styloid process of the radius to record the elbow extensor and flexor movements, respectively. For the assessment of the lower body strength, the participants were seated with their hips and knees flexed at 90° and completed a series of isometric exertions while a dynamometer was held on their leg on the anterior aspect of the shank (proximal to the ankle joint), or on the posterior aspect of the shank (proximal to the ankle joint) to record knee extensor and flexor movements, respectively. The participants gradually increased their effort to maximum and stopped contracting upon instruction by the clinic staff. The movement was repeated after a 15 s rest interval and the values were averaged [23]. The same procedure was repeated on the other side of the body. For the assessment of the handgrip strength, the participants were instructed to hold the dynamometer tightly to their maximum capacity. This procedure was repeated thrice in both the right and left hands, and the average values were calculated as follows:

The upper body strength was calculated by summing the average values of the elbow extension and flexion for both the right and left arms. The lower body strength was calculated by summing the average values of knee extension and flexion for both the right and left legs. The handgrip strength was calculated by summing the average values of both the right and left hands. The composite WBMS was calculated by summing the upper body strength, lower body strength, and handgrip strength.

#### 2.6.2. Endurance Performance

All the participants completed the Modified Bruce protocol treadmill walk test until voluntary exhaustion [24]. The protocol includes nine 3 min stages, during which the speed and gradient of the treadmill were gradually increased with each stage. The endurance performance was assessed by the time on the treadmill and stage achieved during the treadmill walk test.

#### 2.6.3. Body Composition

Weight measurements were recorded using calibrated scales. The waist circumference was measured by fitting the tape measure to the part of the trunk located midway between the lower costal margin and the iliac crest. The other body composition variables such as android/gynoid fat ratio, and fat and muscle mass were measured by using DXA (Lunar Prodigy Advance model).

#### 2.6.4. Questionnaires

The exercise recovery was assessed by the Delayed Onset Muscle Soreness (DOMS) questionnaire, which categorizes muscle soreness on a seven-point scale ranging from no pain to severe pain limiting the ability to move [25]. The DOMS questionnaire was completed electronically 24, 48, and 72 h after the treadmill walk test. The product tolerability and perception were assessed by using the modified Gastrointestinal Symptoms Rating scale (GSRS) and Product Perception Questionnaire, respectively. The modified GSRS consisted of 12 items, each rated on a 4-point scale from no discomfort to severe discomfort. The gastrointestinal symptoms included in the modified GSRS scale were abdominal pain, heartburn, acid regurgitation, nausea and vomiting, borborygmus, abdominal distension, eructation, increased flatus, decreased passage of stools, increased passage of stools, loose stools, and hard stools [26]. The Product Perception Questionnaire, consisting of four questions related to product tolerability, perception, and likeability, were scored on a scale of 0–4, with a higher score indicating a greater level of enjoyment. The quality of life and the general well-being of the participants were assessed by the SF-36 questionnaire, which includes eight scales: physical functioning, role—physical, bodily pain, general health, vitality, social functioning, role—emotional, and mental health. The scores range from 0 to 100 where higher scores indicate a better state of health [27].

#### 2.6.5. Study Diaries and Food Records

The participants completed the study diary daily, which included questions related to the daily product consumption, compliance with the resistance training program, changes in health, adverse events, and concomitant therapies. In addition to the study diaries, the participants used an online food record application called Libro, by Nutritics, to track their food consumption, which was used to calculate the calorie, macronutrient, and micronutrient intake throughout the study. Food records were completed for 3 days (including one weekend day and two weekdays) in the week prior to the clinic visits on days 28, 56, and 84.

#### 2.6.6. Safety

The severity of an AE was classified as “mild”, “moderate”, or “severe”, and the degree of the relationship between the study product and an AE was categorized as “not related”, “unlikely”, “possibly”, “probably”, and “most probably”, by the MD. The hematology parameters included the WBC count with the differential count (neutrophils, lymphocytes, monocytes, eosinophils, basophils), red blood cell (RBC) count, hemoglobin, hematocrit, platelet count, and RBC indices (mean corpuscular volume, mean corpuscular hemoglobin, mean corpuscular hemoglobin concentration, mean platelet volume, and red cell distribution width). The clinical chemistry parameters included liver function (alanine aminotransferase, aspartate aminotransferase, alkaline phosphatase, total bilirubin), kidney function (creatinine, electrolytes (sodium, potassium, and chloride), and estimated glomerular filtration rate), and glucose. All the blood parameters were analyzed by using LifeLabs (London, ON, Canada) using the standardized procedures. The clinical significance of abnormal clinical chemistry and hematology laboratory values was assessed by the MD.

### 2.7. Study Procedures

The participants were instructed to complete the resistance training program throughout the study period (see Section Resistance Training Program). At the baseline and on days 28, 56, and 84, in-clinic assessments for muscle strength and endurance performance were conducted, QoL and product tolerability questionnaires were administered, and blood was drawn for CRP measurements. The assessment of body composition via DXA was conducted at the baseline and day 84, and the Product Perception Questionnaire was administered at day 84. The clinical assessments for vitals were conducted at each clinic visit, with blood drawn for the measurement of the safety blood parameters at screening and at the end of each study visit. The urine pregnancy tests for females with child-bearing potential were completed at the baseline and day 84.

#### Resistance Training Program

The participants were instructed to complete six 30 min sessions of resistance training each week during the study period. Of the six sessions, three focused on the upper body and three focused on the lower body (Table 1). The sessions focusing on the same muscle groups (upper body or lower body) were separated by at least 48 h. The exercises were completed to fatigue using a self-selected moderate amount of resistance. The 30 min sessions consisted of 20 min of resistance exercises and 5 min each of warmup and cooldown low-intensity exercises. The participants were allowed to combine the upper and lower body exercises into one 50 min session (three sessions/week) at their own discretion, consisting of 40 min of resistance exercises and 5 min each of warmup and cooldown exercises. This program was in agreement with the recommendations of the Canadian Society for Exercise Physiology (CSEP) and the American College for Sports Medicine (ACSM) [20,28]. To assess the compliance with the resistance training program, the participants recorded the duration and type of resistance training (upper body, lower body, or both), weight used, number of repetitions, and information on warmup and cooldown in their study diaries. Each participant’s exercise compliance per week (%) was determined by dividing the actual weekly score by the maximum weekly score multiplied by 100. The overall exercise compliance was calculated by averaging the weekly exercise compliance from the baseline to the end of the study.

### 2.8. Statistical Analysis

A sample size of 25 participants per group provided 80% power (considering a two-sided alpha of 0.05 and a 20% assumed attrition rate) to detect the differences in the mean change in handgrip strength (1.36 kg), elbow flexion (6.3 kg), elbow extension (3.7 kg), knee flexion (38 kg), and knee extension (17.6 kg) between the groups [29,30,31].

The summary statistics for the continuous outcome measures at each timepoint and changes from the baseline at each timepoint (28, 56, 84 days) are presented as means, medians, standard deviations, minimum, maximum, and proportions (if categorical). All the primary and secondary outcomes were evaluated for normality. The primary outcome was assessed by using a linear mixed model using untransformed data (normal distribution) or log-transformed data (log-normal distribution), including participant ID as a random effect and study groups, time (visit number), and interaction between visit and group as fixed effects. If an outcome variable was not normally or log-normally distributed, then Friedman’s test on untransformed data was used. The analyses are reported for the Per protocol (PP) population consisting of all the participants who consumed at least 80% of the study product, did not have any major protocol violations related to the primary outcome, and completed all the study visits and procedures connected with the measurement of the primary variable. All the statistical analyses were performed using the R Statistical Software Package Version 3.6.3 or newer for Microsoft Windows [32]. *p* values ≤ 0.05 were considered statistically significant.

## 3. Results

### 3.1. Study Population

**Figure 2:**
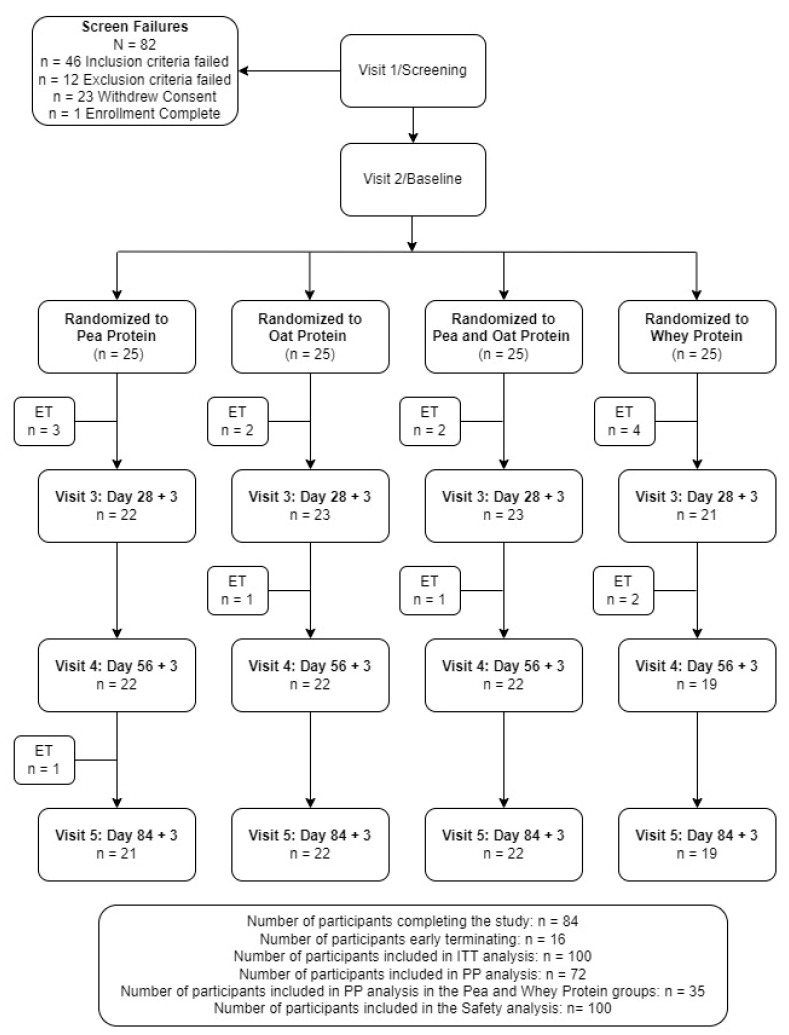
Disposition of study participants. ITT, intention-to-treat. PP, per-protocol.

A total of 182 volunteers were screened with 100 eligible participants consenting and enrolled in the study (Figure 2). Fifteen participants were excluded from the PP analysis due to early terminations (n = 10), out of window study visits (n = 3), and <80% exercise compliance (n = 2), with seven and eight participants excluded from the PPr and WPr groups, respectively. Forty-eight percent of the participants supplemented with PPr or WPr were men, and the average age of the reported population was 40.24 ± 8.20 years. There were no significant differences between the groups in the demographic and anthropometric variables (Table 2). The study product compliance was greater than 98% for both study groups. The overall exercise compliance was 98.76 ± 2.82% and 97.39 ± 6.13% for the PPr and WPr groups (*p* = 0.79), respectively. All the participants were healthy as determined by their medical history, vital signs, hematology, and clinical chemistry parameters as assessed by the MD.

### 3.2. Composite WBMS and Individual Muscle Strength

**Figure 3:**
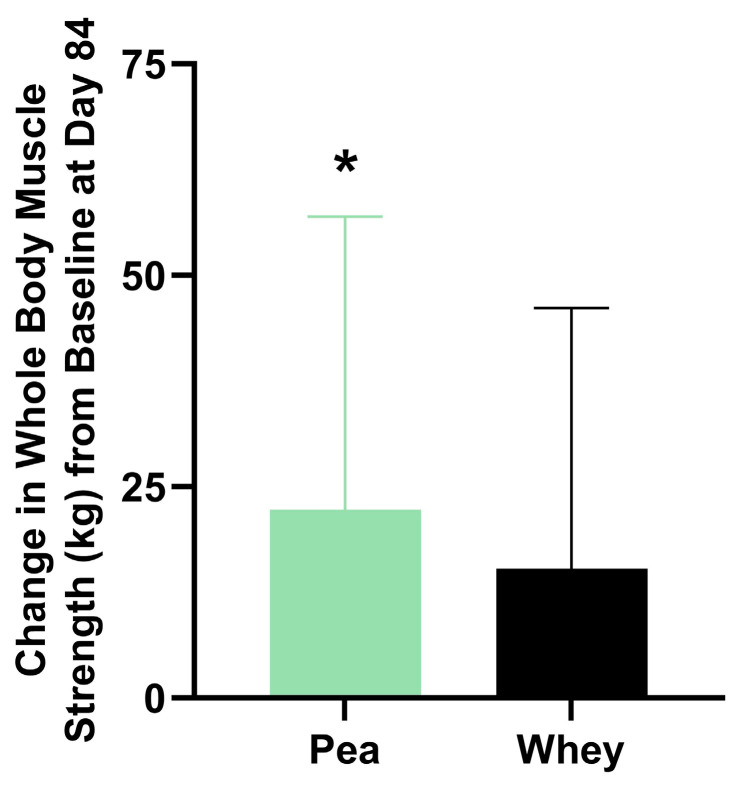
Change in composite whole-body muscle strength from baseline at day 84 in the pea and whey protein groups. * indicates a significant within-group difference in strength from baseline (*p* < 0.05).

**Figure 4:**
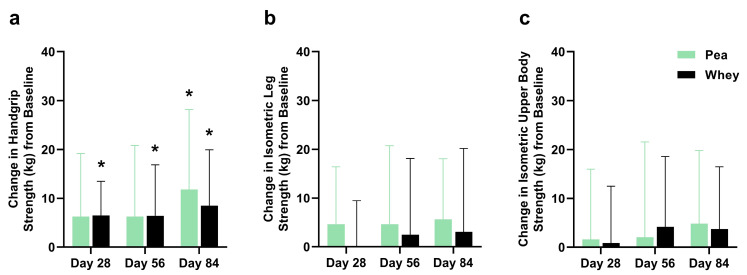
Change in (**a**) handgrip, (**b**) isometric leg, and (**c**) isometric upper body strength from baseline during the study period in the pea and whey protein groups. * indicates a significant within-group difference in strength from baseline at Day 28, 56, or 84 (*p* < 0.05).

There were no significant differences between the groups in their composite WBMS or individual muscle strength. The participants supplemented with PPr for 84 days had a 16.1% improvement in their composite WBMS (*p* = 0.01), while those on WPr had an 11.1% improvement (*p* = 0.06) (Figure 3). The PPr group had an 18.2% improvement in their handgrip strength from the baseline at day 84, while the WPr group had 9.9%, 9.8%, and 13.0% improvements from the baseline at days 28, 56, and 84 (*p* ≤ 0.02) (Figure 4a). The changes in isometric leg strength and upper body strength are presented in Figure 4b,c.

### 3.3. Endurance Performance

Endurance performance, as assessed by the change from the baseline in the total time on the treadmill during the treadmill walk test, was significantly greater in the PPr group at day 28 (0.7 ± 1.1 min) and day 56 (1.1 ± 1.2 min) compared to the WPr group (−0.1 ± 2 min and −0.3 ± 5.8, respectively). However, the significantly greater increase in endurance performance was not sustained at the end of the study (0.69 ± 1.9 min in PPr vs. 0.3 ± 2.2 min in WPr, *p* = 0.14). There were no significant differences between the groups in endurance performance, as assessed by the change from the baseline in the stage achieved during the treadmill walk test. The PPr group had increases of 0.28 ± 0.67, 0.22 ± 0.55, 0.06 ± 0.73 (*p* ≥ 0.11) at days 28, 56, and 84, respectively, while the WPr group had increases of 0.3 ± 0.6, 0.3 ± 1.2, 0.3 ± 0.7 (*p* ≥ 0.06), respectively.

### 3.4. Exercise Recovery

The participants supplemented with PPr had significant improvements in their 24 h (−1.24 ± 2.05), 48 h (−0.89 ± 1.81), and 72 h (−2.06 ± 3.89) post-exercise DOMS score from the baseline at day 28, with significantly greater improvement in their 24 h DOMS score compared to WPr (0.35 ± 2.45) (*p* = 0.05). Further, the PPr group had significant improvements in their 24 h (−1.41 ± 2.35) and 72 h (−1.94 ± 3.98) DOMS score from the baseline at days 56 and 84 (*p* ≤ 0.05), respectively, while the WPr group had improvements in their 48 h (−2 ± 3.37) and 72 h (−2 ± 3.79) DOMS score from the baseline at day 84 (*p* ≤ 0.05).

### 3.5. Body Composition

There were no significant differences between the groups in their body composition measures. The participants supplemented with PPr had a 2.3% improvement in muscle mass from the baseline at day 84 (*p* < 0.01), while those on WPr had an improvement of 2.4% (*p* = 0.01) (Table 3). The PPr group had a decrease of 0.73 ± 1.66% (*p* = 0.08) in fat mass after 84 days of supplementation, while the WPr group had a decrease of 0.81 ± 2.31% (*p* = 0.17). Additionally, the PPr and WPr groups had respective changes in their body weight of −0.1 ± 3.7 kg and 0.6 ± 1.3 kg, and in their waist circumference of −0.7 ± 3.2 cm and −2.2 ± 5.9 cm.

### 3.6. Quality of Life

There were no significant differences between the groups in their quality of life. The participants supplemented with PPr had significantly greater improvements in their role—emotional scores at days 28 and 84 compared to those on WPr (*p* ≤ 0.05). Both the PPr and WPr groups had significant improvements in vitality from the baseline at days 28 (PPr: 8.61 ± 12.1 vs. WPr: 9.71 ± 9.92), 56 (7.5 ± 14.17 vs. 10.59 ± 12.36), and 84 (13.61 ± 14.73 vs. 10.59 ± 12.98) (*p* ≤ 0.04), and significant improvements in their reported health transition from the baseline at days 28 (12.50 ± 19.65 vs. 14.71 ± 23.48), 56 (20.83 ± 17.68 vs. 16.18 ± 21.54), and 84 (18.06 ± 18.80 vs. 19.12 ± 24.25) (*p* ≤ 0.02). Further, the PPr group had significant improvements in role—physical (13.89 ± 26.04), general health (6.67 ± 7.48), mental health (5.78 ± 10.1), and physical functioning (5 ± 9.39) from the baseline at day 84 (*p* ≤ 0.04). From the baseline at day 56, the participants supplemented with PPr had significant improvements in their general health (5.83 ± 7.91) and social functioning (6.25 ± 10.72) (*p* ≤ 0.02).

### 3.7. Immune Function

There were no significant between- or within-group differences in immune function as assessed by white blood cell, lymphocyte, and neutrophil counts (Table S2). The C-reactive protein levels at days 28 and 84 were significantly greater in the PPr group compared to those supplemented with WPr. However, the changes in CRP were considered not clinically relevant by the MD (Table S3).

### 3.8. Product Tolerability and Perception

After 84 days of supplementation, the PPr and WPr groups had decreases of 30.5% and 43.1% in their product tolerability scores, as assessed by their modified GSRS scores, indicating the low severity of gastrointestinal symptoms (Table S4). Further, there were no significant differences between the PPr and WPr groups in product tolerability, perception, and likeability at the end of the study (Tables S5–S7).

### 3.9. Dietary Intake

There were no significant differences between the groups in total calories, fat, protein, or carbohydrates (Table S8). The protein intake of the participants was maintained at 1.1 g/kg bw/day and 1.3 g/kg bw/day for the PPr and WPr groups, respectively.

### 3.10. Safety

Supplementation with PPr for 84 days was safe and well tolerated in the population investigated. A total of 23 post-emergent AEs were reported by 14 unique participants: 13 by 7 participants in the PPr group and 10 by 7 participants in the WPr group. No AEs were categorized as “most probable”, “probably”, or “possibly” related to the study product. All the hematology and clinical chemistry values outside the normal laboratory range were deemed not clinically relevant by the MD, except for one participant with an elevated total bilirubin in the PPr group. This participant was lost to follow-up and was advised to contact their general practitioner.

## 4. Discussion

The daily supplementation with PPr or WPr for 84 days in combination with a resistance training program resulted in comparable strength and muscle mass gains. There were no significant differences in product perception or likability, suggesting that the protein products were enjoyed similarly among the participants. Importantly, the PPr supplementation was found to be safe and well tolerated in the population studied. The findings from this study confirm the results from previous investigations that explored the effect of PPr on the strength and recovery outcomes in sedentary and physically active individuals [10,18,19]. In addition to the equitable strength and muscle performance, the consumption of PPr may have widespread environmental and personal health advantages over WPr. Unlike animal-sourced proteins, plant-based protein production requires fewer natural resources, and plant-based foods are known to reduce the lifetime risk of chronic conditions [11]. Furthermore, WPr is a by-product of dairy manufacturing [8], which represents the most common worldwide food allergen. Substituting the consumption of WPr with PPr may mitigate the environmental and personal risks, while maintaining the strength and anthropometric benefits in heterogeneous populations.

The strength and muscle mass improvements attributed to protein supplementation are directly related to the nutritional quality of proteins and the composition of EAAs [33]. Pre-clinical studies report that both WPr and PPr have fast digestion kinetics, meaning that the concentration of amino acids in the blood rapidly increases after ingestion [34] and they are readily available for muscle protein synthesis. The digestible indispensable amino acid score (DIAAS) is a new protein quality measure recommended by the Food and Agriculture Organization (FAO) to assess the composition of indispensable amino acids in a protein and their individual ileal digestibility [35]. The ileal amino acid digestibility value is considered an accurate measure compared to the digestibility value obtained from fecal methods which is impacted by the colonic microbiota metabolizing the residual dietary amino acids [36]. PPr was recently found to have a DIAAS of 1.00, demonstrating its ability to meet the amino acid requirements and provide a source of high-quality protein [37]. The consumption of EAAs in combination with resistance training stimulates the activation of the mammalian target of the rapamycin (mTOR) signaling pathway, leading to muscle hypertrophy [38]. PPr and WPr contain leucine, an essential amino acid that has been shown to promote the muscle protein synthesis and repair, with large effects in young adults [39]. However, the quantity of leucine, and other EAAs, vary greatly between these protein sources. WPr is considered a complete protein as it is composed of all the nine EAAs in adequate quantities for optimal physiological function [40]. Even though PPr also contains all the nine EAAs, it has been considered incomplete due to an inadequate quantity of methionine [41]. Despite the differences between the PPr and WPr amino acid composition, the findings of the current study demonstrated comparable benefits of PPr and WPr supplementation for individual muscle strength and mass. This is supported by the findings of a recent study demonstrating the equivalent rates of muscle protein synthesis following the ingestion of the same amounts of PPr or milk-derived protein [42]. While there were no significant differences between the groups in their composite WBMS, only the PPr group had statistically significant improvements in their composite WBMS after 84 days of supplementation. Large inter-individual variability may have impacted these differences, perhaps most notably the lack of statistically significant improvement in the composite WBMS for those supplemented with WPr. Considering the abundance of evidence demonstrating the benefit of WPr for muscular strength (4), this is a surprising result.

The direct cause of the observed inter-individual variability, whether sourced from differential responses to protein supplementation, resistance training, or both, cannot be confirmed from the study results, but warrant further discussion. The participants in this study were healthy and had a sedentary lifestyle, defined as engaging in less than 60 min of regular and structured moderate to vigorous exercise per week. To limit the confounding effects of exercise and diet, the participants were required to comply with the resistance training program and maintain their daily caloric intake throughout the study. The improvements in their composite WBMS may have been partly attributed to the recruitment of an exercise-naïve population, as other studies have found a greater effect on the muscle mass in participants with lower muscle force at the baseline [19]. Furthermore, the exercise guidelines instituted in this study could be classified as a high-intensity resistance training program, which has been found to impact strength gains to a greater extent compared to low-intensity training [43]. Previous research suggests that the variability in strength and muscle mass-related outcomes may be expected in a sedentary population, with some participants having dramatic increases in muscle mass compared to others [44]. In more homogenous athletic populations, the variability in strength outcomes has been found to be less prominent [10]. The differential responses to resistance training may have been influenced by individual training goals and diet throughout the study period, leading to enhanced improvements for some participants. The amount of weight chosen by the participants during exercise, and their motivation to increase that weight week to week, could have had a large impact on their strength gains. The “repetition continuum” model proposes that heavy loads at low repetitions is optimal for maximal strength, while light loads at high repetitions are best for muscular endurance [45]. Although physical activities of higher intensity are more likely to result in greater health benefits, sedentary individuals may be less compliant with such training programs [43]. Lower training frequency translates to less exercises performed, and ultimately less muscle gained [46].

In the current study, the exercise compliance of the study population ranged from 80.5% to 105.6%, suggesting that the subgroup analyses based on exercise compliance may be warranted. Furthermore, even with 100% compliance with the exercise guidelines, the sedentary behavior of the participants throughout the study period may have varied. Some sedentary time would have been substituted with time spent exercising as part of the normal study procedures, but it is also possible that the participants compensated for their increased exercise by being more sedentary in their other lifestyle domains. This phenomenon, termed compensatory sedentary behavior, may be more prominent in individuals engaging in high-intensity exercise but has also been reported in office employees undergoing considerably less intense sit-stand desk interventions [47]. The reasons for compensatory sedentary behavior include fatigue and the belief that a greater amount of exercise performed once can be balanced with a less amount of exercise later [48]. Depending on the level of compensatory sedentary behavior performed by individuals in this study, the strength, muscle mass, and metabolic improvements may have been diminished [1]. Research has shown that health benefits from 25–35 min of moderate physical activity per day, similar to the amount of exercise prescribed in this study, can be completely negated by corresponding sedentary behavior in a dose-dependent manner [49].

The limitations of the present study should be acknowledged. The amount of protein given to each group in both vanilla and chocolate flavors varied; however, the differences were negligible from a nutrition perspective and were likely overcome by the protein sourced from the regular diet of the participants. Further, although the protein consumption by the participants in each group was not significantly different, the association with muscle function was not investigated in the current study. Considering the relationship between a higher consumption of protein and muscle gains, the observed inter-individual variability in the muscle strength and anthropometric outcomes may have occurred due to the differences in individual dietary protein. The variability in muscle strength may have also impacted the muscle soreness following the treadmill test; however, this was not analyzed in the current study. Additionally, individual training goals, exercise compliance, and baseline and compensatory sedentary behavior may have contributed to the differential responses to the resistance training. The amount of weight and number of repetitions that could be completed as part of the exercise intervention varied between the participants depending on their individual strength which may have confounded the response to protein supplementation and strength gains. Future studies investigating the effect of protein supplementation on muscle function may consider including a control group as well as evaluating the influence of the overall protein consumption, exercise compliance, and weekly hours of baseline sedentary behavior on muscle function and anthropometric outcomes. As exercise stimulus is an important consideration for strength gains, future studies may consider a progressive overload exercise intervention to further investigate the efficacy of PPr supplementation on muscular performance.

## 5. Conclusions

Eighty-four days of PPr supplementation provided improvements in muscle strength and mass comparable to WPr when combined with a resistance training program. Importantly, PPr supplementation was found to be safe and well tolerated in a population of healthy sedentary adults, with no differences in the incidence of AEs, nor any clinically relevant changes in the clinical chemistry or hematology profiles between WPr and PPr. The results of this study suggest that PPr supplementation paired with a resistance training program may be considered as a viable alternative to WPr without sacrifices in muscular gains or protein product enjoyment, particularly for those concerned with the associated environmental and individual health impacts of animal-sourced proteins.

## Figures and Tables

**Table 1:** Exercises for the resistance training program.

Upper Body Exercises ^1^	Lower Body Exercises ^1^
Chest/Bench press	Leg press
Pushups	Squats
Pec dec	Lunges
Bench press	Deadlifts
Shoulder press	Calf raises
Row	Knee flexion/Leg curl
Back extension	Knee extension/Leg extension
Lat pull down (front or back)	
Bicep curl/arm flexion	
Triceps extension/arm extension	
Abdominal crunch	
Low-Intensity Warmup and Cooldown Exercises	
Walking on the spot, on a treadmill, or outside	
Jogging on the spot, on a treadmill, or outside	
Cycling	

^1^ The participants were asked not to repeat each exercise in more than two sessions per week.

**Table 2:** Baseline demographic characteristics.

Characteristics	Level	Pea	Whey
Age (years)	Mean ± SD Median (Min to Max) (n) Pea vs. Whey *p*-Value *	40.36 ± 8.57 39 (30 to 58) (n = 25) 0.24	40.12 ± 7.82 38 (31 to 56) (n = 25)
Sex	Male	12 (48%)	12 (48%)
	Female	13 (52%)	13 (52%)
	*p*-values **	0.4	0.4
Weight (kg)	Mean ± SD Median (Min to Max) (n) Pea vs. Whey *p*-Value *	75.14 ± 24.12 73.8 (47 to 173) (n = 25) 0.95	71.44 ± 8.4 70.5 (52 to 83.4) (n = 25)
Alcohol Use (n, %)	Ex-drinker	0 (0%)	0 (0%)
	No	9 (36%)	6 (24%)
	Yes	16 (64%)	19 (76%)
	*p*-values **	0.61	0.71
Tobacco Use (n, %)	Ex-smoker	2 (8%)	3 (12%)
	No	21 (84%)	20 (80%)
	Yes	2 (8%)	2 (8%)
	*p*-values **	0.31	0.32

* *p*-values for testing difference between pea and whey were generated by the two-sample *t*-test; ** test of independence using Chi-square test.

**Table 3:** Muscle mass at baseline and day 84.

Muscle Mass (kg)	Pea	Whey	Between Group *p*-Value *
Baseline			
Mean ± SD	44.95 ± 9.73	45.04 ± 9.20	0.98
Median (Min to Max)	44.35 (27.77 to 60.33)	43.95 (30.55 to 62.51)	
n	18	17	
Day 84			
Mean ± SD	46.0 ± 9.89	46.13 ± 9.46	0.97
Median (Min to Max)	45.85 (29.57 to 60.80)	44.38 (32.76 to 61.55)	
n	18	17	
Change from Baseline at Day 84 **			
Mean ± SD	1.05 ± 1.09	1.09 ± 1.59	0.92
Median (Min to Max)	1.09 (−0.64 to 3.50)	0.71 (−0.96 to 5.62)	
n	18	17	
Within Group *p*-value	0.001	0.01	

* *p*-values for testing difference between pea and whey were generated by the two-sample *t*-test. ** *p*-values for testing change from baseline were generated by the paired *t*-test.

## Data Availability

The data presented in this study are available on request from the corresponding author due to privacy and ethical restrictions.

## Supplementary Materials

The following supporting information can be downloaded at: https://www.mdpi.com/article/10.3390/nu16132017/s1, Table S1: CONSORT 2010 checklist of information to include when reporting a randomized trial; Table S2. Immune function variables; Table S3. Blood CRP concentrations (mg/L); Table S4. Product tolerability as assessed by the modified Gastrointestinal Symptoms Rating Scale (GSRS); Table S5. Product tolerability as assessed by the Product Perception Questionnaire; Table S6. Product likeability as assessed by the Product Perception Questionnaire; Table S7. Product perception as assessed by the Product Perception Questionnaire; Table S8. Calorie and macronutrient intake during the study period.
