# Supplementary material for: Efficacy of Pea Protein Supplementation in Combination with a Resistance Training Program on Muscle Performance in a Sedentary Adult Population: A Randomized, Comparator-Controlled, Parallel Clinical Trial
Source: Nutrients. 2024 Jun 26;16(13):2017. doi: 10.3390/nu16132017 (PMC11243455; doi:10.3390/nu16132017)
Supplement: Supplementary file 1 [file nutrients-16-02017-s001.zip › nutrients-3038460-supplementary.pdf]

Table S1. CONSORT 2010 checklist of information to include when reporting a randomised trial*

Section/Topic	Item No	Checklist item	Reported on page No
**Title and abstract**			
	1a	Identification as a randomised trial in the title	1
	1b	Structured summary of trial design, methods, results, and conclusions (for specific guidance see CONSORT for abstracts)	1
**Introduction**			
Background and objectives	2a	Scientific background and explanation of rationale	1-2
	2b	Specific objectives or hypotheses	2
**Methods**			
Trial design	3a	Description of trial design (such as parallel, factorial) including allocation ratio	2
	3b	Important changes to methods after trial commencement (such as eligibility criteria), with reasons	NA
Participants	4a	Eligibility criteria for participants	3
	4b	Settings and locations where the data were collected	2
Interventions	5	The interventions for each group with sufficient details to allow replication, including how and when they were actually administered	3
Outcomes	6a	Completely defined pre-specified primary and secondary outcome measures, including how and when they were assessed	4
	6b	Any changes to trial outcomes after the trial commenced, with reasons	NA
Sample size	7a	How sample size was determined	7
	7b	When applicable, explanation of any interim analyses and stopping guidelines	NA
Randomisation:			
Sequence generation	8a	Method used to generate the random allocation sequence	4
	8b	Type of randomisation; details of any restriction (such as blocking and block size)	4

Allocation concealment mechanism	9	Mechanism used to implement the random allocation sequence (such as sequentially numbered containers), describing any steps taken to conceal the sequence until interventions were assigned	4
Implementation	10	Who generated the random allocation sequence, who enrolled participants, and who assigned participants to interventions	4
Blinding	11a	If done, who was blinded after assignment to interventions (for example, participants, care providers, those assessing outcomes) and how	4
	11b	If relevant, description of the similarity of interventions	4
Statistical methods	12a	Statistical methods used to compare groups for primary and secondary outcomes	7
	12b	Methods for additional analyses, such as subgroup analyses and adjusted analyses	NA
**Results**			
Participant flow (a diagram is strongly recommended)	13a	For each group, the numbers of participants who were randomly assigned, received intended treatment, and were analysed for the primary outcome	7
	13b	For each group, losses and exclusions after randomisation, together with reasons	Figure 2
Recruitment	14a	Dates defining the periods of recruitment and follow-up	2
	14b	Why the trial ended or was stopped	NA
Baseline data	15	A table showing baseline demographic and clinical characteristics for each group	Table 2
Numbers analysed	16	For each group, number of participants (denominator) included in each analysis and whether the analysis was by original assigned groups	7
Outcomes and estimation	17a	For each primary and secondary outcome, results for each group, and the estimated effect size and its precision (such as 95% confidence interval)	7-12
	17b	For binary outcomes, presentation of both absolute and relative effect sizes is recommended	NA
Ancillary analyses	18	Results of any other analyses performed, including subgroup analyses and adjusted analyses, distinguishing pre-specified from exploratory	NA
Harms	19	All important harms or unintended effects in each group (for specific guidance see CONSORT for harms)	12

**Discussion**			
Limitations	20	Trial limitations, addressing sources of potential bias, imprecision, and, if relevant, multiplicity of analyses	14
Generalisability	21	Generalisability (external validity, applicability) of the trial findings	12-14
Interpretation	22	Interpretation consistent with results, balancing benefits and harms, and considering other relevant evidence	12-14
**Other information**			
Registration	23	Registration number and name of trial registry	2
Protocol	24	Where the full trial protocol can be accessed, if available	2
Funding	25	Sources of funding and other support (such as supply of drugs), role of funders	14

Citation: Schulz KF, Altman DG, Moher D, for the CONSORT Group. CONSORT 2010 Statement: updated guidelines for reporting parallel group randomised trials. BMC Medicine. 2010;8:18.

© 2010 Schulz et al. This is an Open Access article distributed under the terms of the Creative Commons Attribution License (http://creativecommons.org/licenses/by/2.0), which permits unrestricted use, distribution, and reproduction in any medium, provided the original work is properly cited.

*We strongly recommend reading this statement in conjunction with the CONSORT 2010 Explanation and Elaboration for important clarifications on all the items. If relevant, we also recommend reading CONSORT extensions for cluster randomised trials, non-inferiority and equivalence trials, non-pharmacological treatments, herbal interventions, and pragmatic trials. Additional extensions are forthcoming: for those and for up-to-date references relevant to this checklist, see www.consort-statement.org

Table S2. Immune function variables

Variables	Group	Screening	Day 84	Change from Screening at Day 84
White Blood Cell Count (x10^9/L)	**Pea**			
	Mean ± SD	6.3 ± 1.7	6.6 ± 1.52	-0.06 ± 1.18
	Median (Min to Max)	5.9 (4.3 to 11.7)	6 (3.9 to 10)	0 (-2.8 to 1.9)
	(n)	(n = 18)	(n = 18)	(n = 18)
	Pea Vs Whey P-Value*	0.35	0.31	0.40
	Within Group P-Value**			0.83
	**Whey**			
	Mean ± SD	5.8 ± 1.4	6.1 ± 1.35	0.3 ± 1.33
	Median (Min to Max)	5.6 (3.8 to 8.8)	6.1 (4.1 to 8.5)	0.2 (-1.8 to 3.3)
	(n)	(n = 17)	(n = 17)	(n = 17)
	Within Group P-Value**			0.37
Absolute Neutrophils (x10^9/L)	**Pea**			
	Mean ± SD	3.62 ± 1.47	3.79 ± 1.19	0.17 ± 1.02
	Median (Min to Max)	3.4 (1.7 to 8.8)	3.6 (1.9 to 6.4)	0.1 (-2.4 to 1.5)
	(n)	(n = 18)	(n = 18)	(n = 18)
	Pea Vs Whey P-Value*	0.64	0.74	0.80
	Within Group P-Value**			0.49
	**Whey**			
	Mean ± SD	3.4 ± 1.26	3.66 ± 1.1	0.26 ± 1.05
	Median (Min to Max)	3.3 (1.4 to 6)	3.4 (1.9 to 5.5)	0.3 (-0.9 to 3.3)
	(n)	(n = 17)	(n = 17)	(n = 17)
	Within Group P-Value**			0.32
Absolute Lymphocytes (x10^9/L)	**Pea**			
	Mean ± SD	2.07 ± 0.75	1.9 ± 0.53	-0.17 ± 0.6
	Median (Min to Max)	2 (1.2 to 4.8)	1.8 (1.3 to 3)	-0.1 (-2.6 to 0.3)
	(n)	(n = 18)	(n = 18)	(n = 18)
	Pea Vs Whey P-Value*	0.14	0.27	0.62
	Within Group P-Value**			0.25
	**Whey**			
	Mean ± SD	1.76 ± 0.39	1.69 ± 0.57	-0.07 ± 0.58
	Median (Min to Max)	1.7 (1.2 to 2.7)	1.7 (0.3 to 2.6)	0 (-1.8 to 0.7)
	(n)	(n = 17)	(n = 17)	(n = 17)
	Within Group P-Value**			0.63

*P-values for testing difference between Pea vs. Whey were generated by the two-sample t-test. **P-values for testing change from baseline were generated by the paired t-test.

Table S3. Blood CRP concentrations (mg/L)

Group	Baseline	Day 28	Day 56	Day 84	Change from Baseline at Day 28	Change from Baseline at Day 56	Change from Baseline at Day 84
**Pea**							
Mean ± SD	1.43 ± 1.41	1.34 ± 0.75	1.36 ± 0.85	1.53 ± 1.08	-0.08 ± 0.97	-0.07 ± 0.83	0.1 ± 0.84
Median (Min to Max)	0.9 (0.6 to 6.4)	1.1 (0.6 to 3)	1.15 (0.6 to 3.7)	1.2 (0.6 to 4)	0.05 (-3 to 1.3)	.05 (-2 to 1.5)	0.1 (-1.7 to 2.3)
(n)	(n = 18)	(n = 18)	(n = 18)	(n = 18)	(n = 18)	(n = 18)	(n = 18)
Pea vs. Whey P-Value*	0.12	**0.02**	0.65	**0.02**	0.81	0.15	0.61
Within Group P-Value**					0.73	0.73	0.62
**Whey**							
Mean ± SD	0.86 ± 0.46	0.84 ± 0.36	1.2 ± 1.17	0.85 ± 0.31	-0.02 ± 0.35	0.34 ± 0.81	-0.02 ± 0.5
Median (Min to Max)	0.6 (0.6 to 2.2)	0.6 (0.6 to 1.9)	0.8 (0.6 to 5.3)	0.7 (0.6 to 1)	0 (-0.9 to 0.4)	0 (-0.4 to 3.1)	0 (-1.3 to 1)
(n)	(n = 17)	(n = 17)	(n = 17)	(n = 17)	(n = 17)	(n = 17)	(n = 17)
Within Group P-Value**					0.82	0.10	0.87

*P-values for testing difference between Pea vs. Whey were generated by the two-sample t-test. **P-values for testing change from baseline were generated by the paired t-test.

Table S4. Product tolerability as assessed by Modified Gastrointestinal Symptoms Rating Scale (GSRS)

Group	Baseline	Day 28	Day 56	Day 84	Change from Baseline at Day 28	Change from Baseline at Day 56	Change from Baseline at Day 84
**Total GSRS Score**							
**Pea**							
Mean ± SD	2 ± 2.38	2.61 ± 3.42	1.78 ± 3.95	1.39 ± 3.31	0.61 ± 2.57	-0.22 ± 3.02	-0.61 ± 2.66
Median (Min to Max)	1 (0 to 8)	1 (0 to 11)	0.5 (0 to 17)	0 (0 to 14)	0 (-5 to 6)	0 (-6 to 9)	0 (-6 to 6)
(n)	(n = 18)	(n = 18)	(n = 18)	(n = 18)	(n = 18)	(n = 18)	(n = 18)
Pea Vs Whey P-Value*	0.35	0.23	0.73	0.64	**0.04**	0.21	0.47
Within Group P-Value**					0.33	0.76	0.34
**Whey**							
Mean ± SD	2.88 ± 3.1	1.47 ± 1.7	1.41 ± 2.06	1.65 ± 2.09	-1.41 ± 3.06	-1.47 ± 2.7	-1.24 ± 2.46
Median (Min to Max)	2 (0 to 9)	1 (0 to 6)	1 (0 to 7)	1 (0 to 7)	0 (-9 to 3)	0 (-8 to 2)	0 (-6 to 3)
(n)	(n = 17)	(n = 17)	(n = 17)	(n = 17)	(n = 17)	(n = 17)	(n = 17)
Within Group P-Value**					0.08	**0.05**	**0.05**
**Abdominal Pains**							
**Pea**							
Mean ± SD	0.11 ± 0.32	0 ± 0	0.06 ± 0.24	0.06 ± 0.24	-0.11 ± 0.32	-0.06 ± 0.42	-0.06 ± 0.42
Median (Min to Max)	0 (0 to 1)	0 (0 to 0)	0 (0 to 1)	0 (0 to 1)	0 (-1 to 0)	0 (-1 to 1)	0 (-1 to 1)
(n)	(n = 18)	(n = 18)	(n = 18)	(n = 18)	(n = 18)	(n = 18)	(n = 18)
Pea Vs Whey P-Value*	0.61	1	0.31	0.31	0.61	1	1
Within Group P-Value**					0.16	0.55	0.55
**Whey**							
Mean ± SD	0.06 ± 0.24	0 ± 0	0 ± 0	0 ± 0	-0.06 ± 0.24	-0.06 ± 0.24	-0.06 ± 0.24
Median (Min to Max)	0 (0 to 1)	0 (0 to 0)	0 (0 to 0)	0 (0 to 0)	0 (-1 to 0)	0 (-1 to 0)	0 (-1 to 0)
(n)	(n = 17)	(n = 17)	(n = 17)	(n = 17)	(n = 17)	(n = 17)	(n = 17)
Within Group P-Value**					0.32	0.32	0.32
**Heartburn**							
**Pea**							
Mean ± SD	0.22 ± 0.55	0.33 ± 0.77	0.17 ± 0.51	0.11 ± 0.47	0.11 ± 0.47	-0.06 ± 0.24	-0.11 ± 0.32
Median (Min to Max)	0 (0 to 2)	0 (0 to 3)	0 (0 to 2)	0 (0 to 2)	0 (-1 to 1)	0 (-1 to 0)	0 (-1 to 0)

Group	Baseline	Day 28	Day 56	Day 84	Change from Baseline at Day 28	Change from Baseline at Day 56	Change from Baseline at Day 84
(n)	(n = 18)	(n = 18)	(n = 18)	(n = 18)	(n = 18)	(n = 18)	(n = 18)
Pea Vs Whey P-Value*	0.52	0.18	0.95	0.64	0.19	0.15	0.109
Within Group P-Value**					0.34	0.30	0.16
**Whey**							
Mean ± SD	0.12 ± 0.33	0.06 ± 0.24	0.18 ± 0.39	0.18 ± 0.39	-0.06 ± 0.24	0.06 ± 0.24	0.06 ± 0.24
Median (Min to Max)	0 (0 to 1)	0 (0 to 1)	0 (0 to 1)	0 (0 to 1)	0 (-1 to 0)	0 (0 to 1)	0 (0 to 1)
(n)	(n = 17)	(n = 17)	(n = 17)	(n = 17)	(n = 17)	(n = 17)	(n = 17)
Within Group P-Value**					0.32	0.32	0.32
**Acid Regurgitation**							
**Pea**							
Mean ± SD	0.06 ± 0.24	0.11 ± 0.32	0.11 ± 0.32	0.06 ± 0.24	0.06 ± 0.24	0.06 ± 0.24	0 ± 0
Median (Min to Max)	0 (0 to 1)	0 (0 to 1)	0 (0 to 1)	0 (0 to 1)	0 (0 to 1)	0 (0 to 1)	0 (0 to 0)
(n)	(n = 18)	(n = 18)	(n = 18)	(n = 18)	(n = 18)	(n = 18)	(n = 18)
Pea Vs Whey P-Value*	0.28	0.61	0.92	0.54	0.07	0.15	0.30
Within Group P-Value**					0.30	0.30	1
**Whey**							
Mean ± SD	0.18 ± 0.39	0.06 ± 0.24	0.12 ± 0.33	0.12 ± 0.33	-0.12 ± 0.33	-0.06 ± 0.24	-0.06 ± 0.24
Median (Min to Max)	0 (0 to 1)	0 (0 to 1)	0 (0 to 1)	0 (0 to 1)	0 (-1 to 0)	0 (-1 to 0)	0 (-1 to 0)
(n)	(n = 17)	(n = 17)	(n = 17)	(n = 17)	(n = 17)	(n = 17)	(n = 17)
Within Group P-Value**					0.15	0.32	0.32
**Nausea and Vomiting**							
**Pea**							
Mean ± SD	0 ± 0	0.06 ± 0.24	0 ± 0	0 ± 0	0.06 ± 0.24	0 ± 0	0 ± 0
Median (Min to Max)	0 (0 to 0)	0 (0 to 1)	0 (0 to 0)	0 (0 to 0)	0 (0 to 1)	0 (0 to 0)	0 (0 to 0)
(n)	(n = 18)	(n = 18)	(n = 18)	(n = 18)	(n = 18)	(n = 18)	(n = 18)
Pea Vs Whey P-Value*	1	0.31	1	1	0.31	1	1
Within Group P-Value**					0.30	1	1
**Whey**							
Mean ± SD	0 ± 0	0 ± 0	0 ± 0	0 ± 0	0 ± 0	0 ± 0	0 ± 0
Median (Min to Max)	0 (0 to 0)	0 (0 to 0)	0 (0 to 0)	0 (0 to 0)	0 (0 to 0)	0 (0 to 0)	0 (0 to 0)

Group	Baseline	Day 28	Day 56	Day 84	Change from Baseline at Day 28	Change from Baseline at Day 56	Change from Baseline at Day 84
(n)	(n = 17)	(n = 17)	(n = 17)	(n = 17)	(n = 17)	(n = 17)	(n = 17)
Within Group P-Value**					1	1	1
**Borborygmus**							
**Pea**							
Mean ± SD	0.11 ± 0.32	0.17 ± 0.38	0.17 ± 0.71	0.06 ± 0.24	0.06 ± 0.42	0.06 ± 0.54	-0.06 ± 0.24
Median (Min to Max)	0 (0 to 1)	0 (0 to 1)	0 (0 to 3)	0 (0 to 1)	0 (-1 to 1)	0 (-1 to 2)	0 (-1 to 0)
(n)	(n = 18)	(n = 18)	(n = 18)	(n = 18)	(n = 18)	(n = 18)	(n = 18)
Pea Vs Whey P-Value*	0.47	0.32	0.96	0.54	0.17	0.70	1
Within Group P-Value**					0.55	0.64	0.30
**Whey**							
Mean ± SD	0.18 ±0.39	0.06 ± 0.24	0.18 ± 0.39	0.12 ± 0.33	-0.12 ± 0.33	0 ± 0.35	-0.06 ± 0.24
Median (Min to Max)	0 (0 to 1)	0 (0 to 1)	0 (0 to 1)	0 (0 to 1)	0 (-1 to 0)	0 (-1 to 1)	0 (-1 to 0)
(n)	(n = 17)	(n = 17)	(n = 17)	(n = 17)	(n = 17)	(n = 17)	(n = 17)
Within Group P-Value**					0.15	1	0.32
**Abdominal Distension**							
**Pea**							
Mean ± SD	0.22 ± 0.43	0.22 ± 0.55	0.17 ± 0.51	0.17 ± 0.71	0 ± 0.59	-0.06 ± 0.42	-0.06 ± 0.64
Median (Min to Max)	0 (0 to 1)	0 (0 to 2)	0 (0 to 2)	0 (0 to 3)	0 (-1 to 1)	0 (-1 to 1)	0 (-1 to 2)
(n)	(n = 18)	(n = 18)	(n = 18)	(n = 18)	(n = 18)	(n = 18)	(n = 18)
Pea Vs Whey P-Value*	0.47	0.69	0.48	0.56	0.79	1	0.76
Within Group P-Value**					1	0.55	0.70
**Whey**							
Mean ± SD	0.35 ± 0.61	0.29 ± 0.47	0.29 ± 0.47	0.24 ± 0.44	-0.06 ± 0.75	-0.06 ± 0.56	-0.12 ± 0.49
Median (Min to Max)	0 (0 to 2)	0 (0 to 1)	0 (0 to 1)	0 (0 to 1)	0 (-2 to 1)	0 (-1 to 1)	0 (-1 to 1)
(n)	(n = 17)	(n = 17)	(n = 17)	(n = 17)	(n = 17)	(n = 17)	(n = 17)
Within Group P-Value**					0.75	0.67	0.33
**Eructation**							
**Pea**							
Mean ± SD	0.06 ± 0.24	0.11 ± 0.47	0.11 ± 0.47	0.06 ± 0.24	0.06 ± 0.24	0.06 ± 0.24	0 ± 0
Median (Min to Max)	0 (0 to 1)	0 (0 to 2)	0 (0 to 2)	0 (0 to 1)	0 (0 to 1)	0 (0 to 1)	0 (0 to 0)

Group	Baseline	Day 28	Day 56	Day 84	Change from Baseline at Day 28	Change from Baseline at Day 56	Change from Baseline at Day 84
(n)	(n = 18)	(n = 18)	(n = 18)	(n = 18)	(n = 18)	(n = 18)	(n = 18)
Pea Vs Whey P-Value*	0.42	0.34	0.70	0.54	**0.03**	0.07	<0.001
Within Group P-Value**					0.30	0.30	<0.001
**Whey**							
Mean ± SD	0.18 ± 0.39	0 ± 0	0.06 ± 0.24	0.12 ± 0.33	-0.18 ± 0.39	-0.12 ± 0.33	-0.06 ± 0.24
Median (Min to Max)	0 (0 to 1)	0 (0 to 0)	0 (0 to 1)	0 (0 to 1)	0 (-1 to 0)	0 (-1 to 0)	0 (-1 to 0)
(n)	(n = 17)	(n = 17)	(n = 17)	(n = 17)	(n = 17)	(n = 17)	(n = 17)
Within Group P-Value**					0.08	0.15	0.32
**Increased Flatus**							
**Pea**							
Mean ± SD	0.17 ± 0.38	0.11 ± 0.32	0.22 ± 0.55	0.11 ± 0.32	-0.06 ± 0.24	0.06 ± 0.24	-0.06 ± 0.24
Median (Min to Max)	0 (0 to 1)	0 (0 to 1)	0 (0 to 2)	0 (0 to 1)	0 (-1 to 0)	0 (0 to 1)	0 (-1 to 0)
(n)	(n = 18)	(n = 18)	(n = 18)	(n = 18)	(n = 18)	(n = 18)	(n = 18)
Pea Vs Whey P-Value*	0.67	0.93	0.28	0.57	0.65	**0.03**	1
Within Group P-Value**					0.30	0.30	0.30
**Whey**							
Mean ± SD	0.24 ± 0.56	0.12 ± 0.33	0.06 ± 0.24	0.18 ± 0.39	-0.12 ± 0.49	-0.18 ± 0.39	-0.06 ± 0.66
Median (Min to Max)	0 (0 to 2)	0 (0 to 1)	0 (0 to 1)	0 (0 to 1)	0 (-1 to 1)	0 (-1 to 0)	0 (-2 to 1)
(n)	(n = 17)	(n = 17)	(n = 17)	(n = 17)	(n = 17)	(n = 17)	(n = 17)
Within Group P-Value**					0.33	0.08	0.71
**Decreased Passage of Stools**							
**Pea**							
Mean ± SD	0.56 ± 1.15	0.72 ± 1.27	0.22 ± 0.73	0.33 ± 0.77	0.17 ± 1.25	-0.33 ± 1.41	-0.22 ± 1.35
Median (Min to Max)	0 (0 to 3)	0 (0 to 3)	0 (0 to 3)	0 (0 to 3)	0 (-3 to 3)	0 (-3 to 3)	0 (-3 to 3)
(n)	(n = 18)	(n = 18)	(n = 18)	(n = 18)	(n = 18)	(n = 18)	(n = 18)
Pea Vs Whey P-Value*	0.47	0.43	0.66	0.79	0.18	0.70	0.57
Within Group P-Value**					0.58	0.34	0.50
**Whey**							
Mean ± SD	0.88 ± 1.41	0.41 ± 1	0.35 ± 1	0.41 ± 1	-0.47 ± 1.5	-0.53 ± 1.59	-0.47 ± 1.23
Median (Min to Max)	0 (0 to 3)	0 (0 to 3)	0 (0 to 3)	0 (0 to 3)	0 (-3 to 3)	0 (-3 to 3)	0 (-3 to 1)

Group	Baseline	Day 28	Day 56	Day 84	Change from Baseline at Day 28	Change from Baseline at Day 56	Change from Baseline at Day 84
(n)	(n = 17)	(n = 17)	(n = 17)	(n = 17)	(n = 17)	(n = 17)	(n = 17)
Within Group P-Value**					0.22	0.19	0.14
**Increased Passage of Stools**							
**Pea**							
Mean ± SD	0.17 ± 0.71	0.44 ± 1.04	0.22 ± 0.65	0.22 ± 0.65	0.28 ± 1.32	0.06 ± 1	0.06 ± 1
Median (Min to Max)	0 (0 to 3)	0 (0 to 3)	0 (0 to 2)	0 (0 to 2)	0 (-3 to 3)	0 (-3 to 2)	0 (-3 to 2)
(n)	(n = 18)	(n = 18)	(n = 18)	(n = 18)	(n = 18)	(n = 18)	(n = 18)
Pea Vs Whey P-Value*	0.33	0.40	0.17	0.61	0.14	0.14	0.28
Within Group P-Value**					0.38	0.80	0.80
**Whey**							
Mean ± SD	0.47 ± 1.07	0.18 ± 0.73	0 ± 0	0.12 ± 0.49	-0.29 ± 0.85	-0.47 ± 1.07	-0.35 ± 1.22
Median (Min to Max)	0 (0 to 3)	0 (0 to 3)	0 (0 to 0)	0 (0 to 2)	0 (-3 to 0)	0 (-3 to 0)	0 (-3 to 2)
(n)	(n = 17)	(n = 17)	(n = 17)	(n = 17)	(n = 17)	(n = 17)	(n = 17)
Within Group P-Value**					0.18	0.09	0.25
**Loose Stools**							
**Pea**							
Mean ± SD	0.17 ± 0.38	0.22 ± 0.43	0.28 ± 0.46	0.11 ± 0.32	0.06 ± 0.24	0.11 ± 0.47	-0.06 ± 0.42
Median (Min to Max)	0 (0 to 1)	0 (0 to 1)	0 (0 to 1)	0 (0 to 1)	0 (0 to 1)	0 (-1 to 1)	0 (-1 to 1)
(n)	(n = 18)	(n = 18)	(n = 18)	(n = 18)	(n = 18)	(n = 18)	(n = 18)
Pea Vs Whey P-Value*	0.94	0.89	0.25	0.61	1	0.27	0.70
Within Group P-Value**					0.30	0.34	0.55
**Whey**							
Mean ± SD	0.18 ± 0.39	0.24 ± 0.44	0.12 ± 0.33	0.06 ± 0.24	0.06 ± 0.43	-0.06 ± 0.43	-0.12 ± 0.49
Median (Min to Max)	0 (0 to 1)	0 (0 to 1)	0 (0 to 1)	0 (0 to 1)	0 (-1 to 1)	0 (-1 to 1)	0 (-1 to 1)
(n)	(n = 17)	(n = 17)	(n = 17)	(n = 17)	(n = 17)	(n = 17)	(n = 17)
Within Group P-Value**					0.57	0.57	0.33
**Hard Stools**							
**Pea**							
Mean ± SD	0.17 ± 0.51	0.11 ± 0.47	0.06 ± 0.24	0.11 ± 0.32	-0.06 ± 0.24	-0.11 ± 0.32	-0.06 ± 0.42
Median (Min to Max)	0 (0 to 2)	0 (0 to 2)	0 (0 to 1)	0 (0 to 1)	0 (-1 to 0)	0 (-1 to 0)	0 (-1 to 1)

Group	Baseline	Day 28	Day 56	Day 84	Change from Baseline at Day 28	Change from Baseline at Day 56	Change from Baseline at Day 84
(n)	(n = 18)	(n = 18)	(n = 18)	(n = 18)	(n = 18)	(n = 18)	(n = 18)
Pea Vs Whey P-Value*	0.42	0.70	1	0.93	0.56	0.34	0.41
Within Group P-Value**					0.30	0.16	0.55
**Whey**							
Mean ± SD	0.06 ± 0.24	0.06 ± 0.24	0.06 ± 0.24	0.12 ± 0.33	0 ± 0.35	0 ± 0.35	0.06 ± 0.43
Median (Min to Max)	0 (0 to 1)	0 (0 to 1)	0 (0 to 1)	0 (0 to 1)	0 (-1 to 1)	0 (-1 to 1)	0 (-1 to 1)
(n)	(n = 17)	(n = 17)	(n = 17)	(n = 17)	(n = 17)	(n = 17)	(n = 17)
Within Group P-Value**					1	1	0.57

*P-values for testing difference between Pea vs. Whey were generated by the two-sample t-test. **P-values for testing change from baseline were generated by the paired t-test.

Table S5. Product tolerability as assessed by the Product Perception Questionnaire

Group	Day 84
**Pea**	
Mean ± SD	3.17 ± 1.00
Median (Min to Max)	3 (0.5 to 4)
(n)	(n = 18)
Pea Vs Whey P-Value*	0.88
**Whey**	
Mean ± SD	3.12 ± 0.84
Median (Min to Max)	3 (2 to 4)
(n)	(n = 17)

*P-value for testing difference between Pea vs. Whey was generated by the two-sample t-test.

Table S6. Product likeability as assessed by the Product Perception Questionnaire

Group	Day 84
**Pea**<br>Mean ± SD<br>Median (Min to Max)<br>(n)<br>Pea Vs Whey P-Value*	2.25 ± 1.01<br>2.25 (0.25 to 4)<br>(n = 18)<br>1.00
**Whey**<br>Mean ± SD<br>Median (Min to Max)<br>(n)	2.25 ± 1.14<br>2 (0.5 to 4)<br>(n = 17)

*P-value for testing difference between Pea vs. Whey was generated by the two-sample t-test.

Table S7. Product perception as assessed by the Product Perception Questionnaire

Group	Day 84
**Pea**<br>Mean ± SD<br>Median (Min to Max)<br>(n)<br>Pea Vs Whey P-Value*	2.83 ± 0.87<br>3 (1 to 4)<br>(n = 18)<br>0.19
**Whey**<br>Mean ± SD<br>Median (Min to Max)<br>(n)	2.41 ± 0.97<br>2.5 (0.5 to 4)<br>(n = 17)

*P-value for testing difference between Pea vs. Whey was generated by the two-sample t-test.

Table S8. Calorie and macronutrient intake during the study period

Timepoint	Whey<br>Mean ± SD (n)<br>Median (Min to Max)	Pea<br>Mean ± SD (n)<br>Median (Min to Max)	P-value<br>Estimate [95% CI]
	**Energy (kcal)**		
**V3**	1595.93 ± 502.03 (17)<br>1533.11 (730.64 to 2434.60)	1414.50 ± 458.50 (18)<br>1403.98 (339.30 to 2446.14)	0.272<br>181.43 [-163.70 to 526.55]
**V4**	1790.94 ± 719.77 (17)<br>1623.34 (676.88 to 3354.14)	1523.30 ± 366.27 (18)<br>1514.30 (867.64 to 2459.00)	0.171<br>267.65 [-145.20 to 680.50]
**V5**	1685.30 ± 473.97 (17)<br>1710.26 (892.11 to 2514.00)	1503.44 ± 357.25 (18)<br>1518.68 (805.86 to 2199.52)	0.207<br>181.86 [-120.22 to 483.94]
**Change from V3 to V4**	195.01 ± 614.56 (17)<br>74.47 (-473.03 to 2358.54)	108.79 ± 404.90 (18)<br>78.06 (-478.77 to 1412.98)	0.882 (w)<br>-3.59 [-250.48 to 422.92]
**P-value within group from V3 to V4**	0.174 (w)	0.304 (w)	
**Estimate [95% CI]**	74.47 [-412.63 to 1649.87]	78.06 [-450.90 to 979.81]	
**Change from V4 to V5**	-105.65 ± 821.73 (17)<br>-90.82 (-2462.03 to 1837.12)	-19.86 ± 246.16 (18)<br>-91.07 (-355.94 to 673.48)	0.804 (w)<br>-0.25 [-480.53 to 308.96]
**P-value within group from V4 to V5**	0.517 (w)	0.736	
**Estimate [95% CI]**	-90.82 [-1705.76 to 1253.90]	-19.86 [-339.99 to 494.22]	
**Change from V3 to V5**	89.37 ± 447.22 (17)<br>-10.62 (-527.76 to 1364.10)	88.93 ± 481.21 (18)<br>57.14 (-605.43 to 1664.67)	1.000 (w)<br>-67.76 [-247.40 to 245.34]
**P-value within group from V3 to V5**	0.782 (w)	0.767 (w)	
**Estimate [95% CI]**	-10.62 [-339.14 to 514.46]	57.14 [-217.96 to 373.36]	
	**Carbohydrate (g)**		
**V3**	156.77 ± 60.17 (17)<br>150.37 (71.45 to 274.79)	151.07 ± 50.34 (18)<br>156.79 (38.80 to 260.65)	0.763<br>5.69 [-34.18 to 45.57]

**V4**	178.50 ± 72.68 (17)<br>180.55 (77.11 to 356.53)	163.52 ± 56.69 (18)<br>163.64 (64.26 to 303.34)	0.518<br>14.98 [-28.71 to 58.67]
**V5**	173.58 ± 56.25 (17)<br>173.73 (85.51 to 269.35)	156.53 ± 44.17 (18)<br>153.63 (76.59 to 246.94)	0.324<br>17.05 [-19.33 to 53.43]
**Change from V3 to V4**	21.73 ± 76.48 (17)<br>18.52 (-81.07 to 264.40)	12.45 ± 41.03 (18)<br>3.14 (-50.94 to 125.49)	0.564 (w)<br>15.38 [-30.50 to 49.08]
**P-value within group from V3 to V4**	0.329 (w)	0.215	
**Estimate [95% CI]**	18.52 [-76.03 to 183.14]	12.45 [-42.17 to 91.90]	
**Change from V4 to V5**	-4.92 ± 89.23 (17)<br>1.37 (-258.84 to 125.13)	-6.99 ± 38.28 (18)<br>-13.45 (-65.22 to 82.64)	0.478 (w)<br>14.82 [-42.52 to 46.66]
**P-value within group from V4 to V5**	1.000 (w)	0.449	
**Estimate [95% CI]**	1.37 [-189.39 to 121.73]	-6.99 [-60.30 to 67.41]	
**Change from V3 to V5**	16.81 ± 58.76 (17)<br>16.00 (-93.57 to 134.39)	5.45 ± 57.17 (18)<br>-8.79 (-68.40 to 208.13)	0.228 (w)<br>24.79 [-19.44 to 53.20]
**P-value within group from V3 to V5**	0.255	0.640 (w)	
**Estimate [95% CI]**	16.81 [-11.12 to 44.75]	-8.79 [-29.65 to 38.94]	
	**Protein (g)**		
**V3**	88.68 ± 40.01 (17)<br>76.93 (44.02 to 184.23)	75.25 ± 23.98 (18)<br>75.64 (25.19 to 119.98)	0.680 (w)<br>1.29 [-23.45 to 45.01]
**V4**	95.58 ± 48.44 (17)<br>80.54 (27.00 to 211.27)	76.27 ± 24.16 (18)<br>74.49 (41.47 to 127.74)	0.845<br>19.31 [-8.67 to 45.09]
**V5**	91.27 ± 41.85 (17)<br>78.74 (46.17 to 209.09)	78.44 ± 23.56 (18)<br>81.42 (36.91 to 114.33)	0.912 (w)<br>-2.68 [-20.57 to 21.21]
**Change from V3 to V4**	6.90 ± 24.16 (17)<br>2.08 (-31.03 to 72.72)	1.02 ± 23.77 (18)<br>-3.76 (-33.66 to 50.61)	0.330 (w)<br>5.88 [-9.55 to 21.31]

**P-value within group from V3 to V4**	0.306 (w)	0.858	
**Estimate [95% CI]**	2.08 [-25.62 to 62.03]	1.02 [-32.64 to 47.94]	
**Change from V4 to V5**	-4.31 ± 35.82 (17)<br>-6.06 (-76.23 to 72.85)	2.17 ± 24.94 (18)<br>4.30 (-74.69 to 35.52)	0.255 (w)<br>-10.37 [-26.44 to 13.47]
**P-value within group from V4 to V5**	0.627	0.369 (w)	
**Estimate [95% CI]**	-4.31 [-66.77 to 65.42]	4.30 [-51.26 to 33.41]	
**Change from V3 to V5**	2.59 ± 28.46 (17)<br>-3.51 (-70.08 to 68.71)	3.20 ± 21.34 (18)<br>3.31 (-36.16 to 42.21)	0.779 (w)<br>-6.82 [-21.01 to 18.70]
**P-value within group from V3 to V5**	0.818 (w)	0.534	
**Estimate [95% CI]**	-3.51 [-21.01 to 24.70]	3.21 [-15.79 to 22.16]	
		**Fat (g)**	
**V3**	61.97 ± 20.14 (17)<br>61.70 (12.76 to 98.53)	54.71 ± 24.84 (18)<br>49.32 (8.06 to 111.71)	0.3507<br>7.26 [-8.90 to 23.43]
**V4**	70.46 ± 35.04 (17)<br>60.51 (16.48 to 153.20)	60.24 ± 16.21 (18)<br>57.25 (34.88 to 90.62)	0.271<br>10.23 [-9.52 to 29.98]
**V5**	62.49 ± 27.20 (17)<br>59.75 (13.66 to 136.66)	60.79 ± 22.73 (18)<br>54.39 (27.14 to 108.93)	0.842<br>1.70 [-16.31 to 19.72]
**Change from V3 to V4**	8.49 ± 33.15 (17)<br>-2.43 (-41.10 to 111.61)	5.53 ± 25.57 (18)<br>5.29 (-24.30 to 81.81)	0.961 (w)<br>2.97 [-16.15 to 22.09]
**P-value within group from V3 to V4**	0.854 (w)	0.551 (w)	
**Estimate [95% CI]**	-2.43 [-29.72 to 82.11]	5.29 [-23.18 to 62.73]	
**Change from V4 to V5**	-7.97 ± 46.62 (17)<br>-3.59 (-124.28 to 116.06)	0.55 ± 19.90 (18)<br>-1.11 (-34.89 to 55.70)	0.330 (w)<br>-2.48 [-31.81 to 14.76]

**P-value within group from V4 to V5**	0.244 (w)	0.908	
**Estimate [95% CI]**	-3.59 [-91.23 to 77.40]	0.55 [-30.48 to 40.13]	
**Change from V3 to V5**	0.52 ± 26.61 (17)<br>-1.47 (-48.87 to 74.96)	6.08 ± 30.08 (18)<br>2.79 (-35.87 to 100.87)	0.419 (w)<br>-4.26 [-18.27 to 12.69]
**P-value within group from V3 to V5**	0.678 (w)	0.580 (w)	
**Estimate [95% CI]**	-1.59 [-16.05 to 15.33]	2.79 [-9.65 to 20.81]	

P-values for testing difference between group were generated by the by the two-sample t-test or the Wilcoxon rank sum test, indicated by (w); P-values for testing within group differences were generated by the paired t-test or the Wilcoxon Signed Rank test, indicated by (w).
